# *Pinellia pedatisecta* Schott‐Derived Exosome‐Like Nanovesicles Promote Apoptosis in Colorectal Cancer by Regulating the Lysosome‐Mediated Mitophagy Pathway

**DOI:** 10.1002/fsn3.71500

**Published:** 2026-01-28

**Authors:** Pingsheng Zhou, Qingling Zhang, Xiaotao Zhou, Ge Zhang, Shiping Cheng

**Affiliations:** ^1^ International Education College Jiangxi University of Chinese Medicine Nanchang Jiangxi Province China; ^2^ Graduate School Jiangxi University of Chinese Medicine Nanchang Jiangxi Province China; ^3^ School of Pharmacy Jiangxi University of Chinese Medicine Nanchang Jiangxi Province China; ^4^ Dermatology Department Affiliated Hospital of Jiangxi University of Chinese Medicine Nanchang Jiangxi Province China

**Keywords:** apoptosis, colorectal cancer, exosome‐like nanovesicles, lysosome, mitophagy, *Pinellia pedatisecta* Schott

## Abstract

Plant‐derived exosome‐like nanovesicles (ELNs) have shown potential in the treatment of various diseases. This research sought to investigate the effects of *Pinellia pedatisecta* Schott‐derived ELNs (PPS‐ELNs) on colorectal cancer (CRC). PPS‐ELNs extracted from *Pinellia pedatisecta* Schott were characterized. CRC cell lines HCT116 and HT‐29 were exposed to 10 μg/mL of PPS‐ELNs. Normal colon epithelial cells FHC were treated with different concentrations of PPS‐ELNs. CRC mice were treated with 12.5 or 25 mg/kg of PPS‐ELNs. Subsequent experiments, including cellular uptake assay, CCK‐8 assay, colony formation assay, flow cytometry, Western blot, transmission electron microscopy, LysoTracker Red staining, immunofluorescence, ELISA, in vivo imaging, TUNEL staining, immunohistochemistry, HE staining, and biochemical analysis, were conducted to explore the anti‐CRC effects and potential mechanisms of PPS‐ELNs. Lysosome inhibitor chloroquine was employed to elucidate the underlying mechanism in vitro. The isolated PPS‐ELNs were successfully characterized. Cellular uptake of PPS‐ELNs was observed in CRC cell lines. Notably, PPS‐ELNs did not affect FHC cell viability, while significantly inhibiting proliferation and inducing apoptosis and mitophagy in CRC cell lines. Furthermore, PPS‐ELNs induced oxidative stress and reduced lysosomal damage in HCT116 cells. The effects of PPS‐ELNs on HCT116 cells were reversed by chloroquine. In CRC mice, PPS‐ELNs were primarily accumulated in tumors. PPS‐ELNs markedly reduced tumor growth, induced apoptosis, and decreased Ki67 expression. Additionally, PPS‐ELNs decreased Gal3 expression, increased autophagosomes, and altered mitophagy‐related protein levels in tumor tissues. Importantly, PPS‐ELNs displayed an excellent safety profile in vivo. PPS‐ELNs inhibit CRC progression through the lysosome‐mediated mitophagy pathway.

## Graphical abstract

*Pinellia pedatisecta* Schott‐derived exosome‐like nanovesicles (PPS‐ELNs) are efficiently taken up by colorectal cancer (CRC) cells and exhibit tumor‐targeting properties. Mechanistically, PPS‐ELNs suppress tumor progression by inhibiting the proliferation of CRC cells and promoting apoptosis through regulation of the lysosome‐mediated mitophagy pathway.

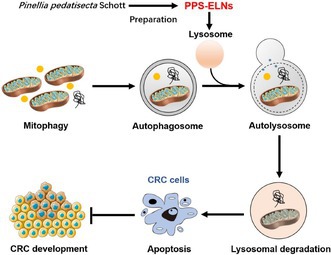

**Abbreviations**
ALT: alanine aminotransferase
AST: aspartate aminotransferase
CQ: chloroquine
CRC: colorectal cancer
ELNs: exosome‐like nanovesicles
PPS: *Pinellia pedatisecta* Schott
PPS‐ELNs: *Pinellia pedatisecta* Schott‐derived ELNs
TCM: traditional Chinese medicine
TME: tumor microenvironment

## 1 Introduction

Colorectal cancer (CRC) is one of the leading cancers worldwide, accounting for 9.6% of new cases (Bray et al. 2024). It is reported that nearly 900,000 individuals die as a result of CRC annually (Dekker et al. 2019). The pathology of CRC involves genetic accumulation and DNA methylation alterations in normal colon epithelium, leading to dysregulation of epithelial cell proliferation and differentiation (Svec et al. 2024; Wang, Ma, et al. 2025). The main risk factors affecting CRC development are age, dietary habits, and inflammatory bowel disease (Gharib and Robichaud 2024). Current therapies include radiotherapy, chemotherapy, surgery, and immunotherapy, which have certain limitations, such as poor efficacy, acquired drug resistance, and postoperative problems (Yu et al. 2024). Thus, developing effective drugs for CRC therapy is necessary.

Lysosomes are key organelles that degrade and recycle impaired proteins and organelles, participate in autophagy and cell death, and are closely related to cancer (Chen et al. 2024). Autophagy is a lysosomal degradation system. In this process, phagocytes form autophagosomes in the cytoplasm, and lysosomes fuse with the autophagosomes and degrade the cytoplasmic substances in them (Fujioka and Noda 2025). Research has shown that Alnustone impedes CRC cell survival by blocking the cell cycle and activating autophagy and apoptosis (Park et al. 2024). Additionally, a previous study revealed the role of lysosomes in resolving TOP1cc DNA damage in vertebrates, while autophagy affects DNA damage repair and cell survival through selective degradation of TOP1cc, which is clinically relevant for CRC patients (Lascaux et al. 2024). Mitophagy is a form of autophagy that maintains cellular homeostasis by clearing damaged mitochondria and regulating genes associated with programmed cell death (Lin et al. 2024). Mitophagy can be induced by multiple interrelated pathways, mainly including PINK1/Parkin‐mediated and receptor‐mediated mitophagy (D'Arcy 2024). Abnormal mitochondrial function has been shown to disrupt cellular activities and induce tumor resistance in CRC (Zhao, Guo, et al. 2024). The GPR176/GNAS complex promotes CRC development by suppressing mitophagy (Tang et al. 2023).

Plant‐derived exosome‐like nanovesicles (ELNs) are a novel natural product, produced through a unique biosynthetic pathway, which mainly involves multivesicular body pathway, exocyst‐positive organelle pathway, and vacuole‐plasma membrane fusion (Song et al. 2025). Plant‐derived ELNs have been extracted from various plants, including *Ganoderma lucidum*, pomegranate, and *Poria cocos*, and demonstrate favorable biological stability and biocompatibility (Kim et al. 2025; Mi et al. 2025; Wang, Zhao, et al. 2025). Therefore, they have gradually become substitutes for mammalian exosomes. *Pinellia pedatisecta* Schott (PPS) serves as a traditional Chinese medicine (TCM) that possesses multiple pharmacological properties, including detumescence, anti‐inflammation, and anti‐tumor effects (Wang et al. 2021). Xiao‐Ban‐Xia decoction, a traditional prescription containing PPS, has been shown to modulate PINK1/Parkin‐mediated mitophagy (Zhao, Han, et al. 2024). Moreover, PPS extract can enhance the function of tumor‐associated dendritic cell antigen presentation and promote an anti‐tumor T cell response (Wang et al. 2019). However, the effects of PPS‐ELNs on CRC remain unclear.

The current research investigated the role of PPS‐ELNs in tumor development in CRC mice and cells. Subsequently, autophagosome formation and the expression of mitophagy pathway‐associated proteins were detected. Finally, CRC cells were treated with the lysosome inhibitor chloroquine (CQ) in the presence of PPS‐ELNs, and it was found that PPS‐ELNs inhibited CRC progression by regulating the lysosome‐mediated mitophagy pathway.

## 2 Methods

### 2.1 Preparation of PPS‐ELNs

The fresh PPS (Bozhou Huqiao Pharmaceutical Co. Ltd., 2,111,260,052) was washed with deionized water and squeezed using a juice extractor. Differential centrifugation was performed on PPS homogenate at 4°C, with centrifugation speeds of 1000, 3000, and 10,000 ***g*** for 10, 20, and 40 min, respectively. The supernatant was ultracentrifuged at 150,000 ***g*** for 90 min, suspended in PBS (Beyotime, C0221A), and filtered through a 0.22 μm membrane. Subsequently, the supernatant was transferred to gradient sucrose solutions (8%, 30%, 45%, and 60%; Yuanyebio, S11055), followed by ultracentrifugation. Visible bands between the 30% and 45% layers were harvested, ultracentrifuged, and filtered to obtain PPS‐ELNs.

### 2.2 Characterization of PPS‐ELNs

To analyze the microscopic morphology of PPS‐ELNs, 10 μL of sample was dropped onto a copper grid and left to settle for 1 min. Subsequently, 10 μL of 2% uranyl acetate (Electron Microscopy Sciences, 22,400) was added and allowed to settle for another 1 min. The dried samples were imaged by transmission electron microscopy (TEM; HITACHI, HT7800) at 100 kV. The particle size and zeta potential of PPS‐ELNs were measured using a Malvern Zetasizer (Bested, 3H‐2000PH).

### 2.3 Assessment of Cellular Uptake of PPS‐ELNs

Sterilized coverslips were placed in a 12‐well plate, and human CRC cell lines HCT116 (Pricella, CL‐0096) and HT‐29 (SunnCell, SNL‐069) were inoculated on the coverslips. Cells were co‐cultured with DiR (Yeasen, 40757ES25)‐labeled PPS‐ELNs (10 μg/mL) for 24 h, and then fixed with 4% paraformaldehyde (Beyotime, P0099). Following this, cells were stained with DAPI (Beyotime, C1005) and observed under laser confocal microscopy (Leica, TCS SP8).

### 2.4 Cell Counting Kit‐8 (CCK‐8)

Cell viability was detected using the CCK‐8 assay kit (Beyotime, C0037). FHC cells (Meisen, CTCC‐001‐0208), HCT116 cells, and HT‐29 cells (2000 cells/well) were seeded into 96‐well plates. After overnight incubation at 37°C with 5% CO_2_, cells were treated with different concentrations of PPS‐ELNs (0, 1, 5, 10, and 50 μg/mL) for 48 h, followed by incubation with 10 μL of CCK‐8 solution for 2 h. Absorbance at 450 nm was measured using a microplate reader (MolecularDevices, SpectraMax M4), and the IC_50_ was determined from the dose–response curve.

### 2.5 Cell Treatment

HCT116 cells were cultured in DMEM (Gibco, C11885500BT) supplemented with 10% fetal bovine serum (Solarbio, S9030), while HT‐29 cells were grown in HT‐29 cell‐specific medium (SunnCell, SNLM‐069). Both cell lines were maintained at 37°C with 5% CO_2_. This experiment consisted of Experiment 1 and Experiment 2. For Experiment 1, HCT116 and HT‐29 cells were divided into control and PPS‐ELNs groups. Cells were treated with 10 μg/mL PPS‐ELNs for 48 h. For Experiment 2, HCT116 cells were divided into control, PPS‐ELNs, and PPS‐ELNs + CQ groups. Cells were simultaneously treated with 10 μg/mL PPS‐ELNs and 10 μM CQ (a lysosome inhibitor, MACKLIN, C798394) for 48 h. Subsequently, cell viability was determined by CCK‐8 assay.

### 2.6 Colony Formation Assay

HCT116 and HT‐29 cells (200 cells/well) were counted and seeded into six‐well plates. After treatment according to the experimental groups, cells were incubated for 7 days, fixed with anhydrous methanol for 15 min, stained with crystal violet (Beyotime, C0121) for 20 min, and photographed.

### 2.7 Flow Cytometry

Cell apoptosis was detected using Annexin V‐FITC apoptosis detection kit (Beyotime, C1086). Cells (5 × 10^4^–1 × 10^5^) were resuspended in 195 μL of Annexin V‐FITC binding solution and incubated with 5 μL of Annexin V‐FITC. Subsequently, cells were incubated with 10 μL of propidium iodide staining solution at room temperature in the dark for 10–20 min. Finally, cells were examined using a flow cytometer (Beckman, CytoFLEX S).

### 2.8 LysoTracker Red Staining

HCT116 cells were inoculated in confocal living cell chambers. After treatment according to grouping, cells were treated with LysoTracker Red working solution (Beyotime, C1046) for 60 min and then incubated with 5 μL Hoechst 33342 staining solution (Beyotime, C1025) for 10 min. Finally, cells were observed under a laser confocal microscope.

### 2.9 Immunofluorescence

Following treatment according to grouping, cells were fixed in 4% paraformaldehyde for 10–15 min and then treated with 1% Triton‐X 100 (Solarbio, T8200) for 5–10 min. After blocking with 3% BSA for 30 min, cells were incubated with primary antibodies, including TOM20 (1:400, Proteintech, 66,777) and LC3 (1:200, Affinity, AF5402), at 4°C overnight. Subsequently, cells were incubated with goat anti‐mouse IgG (1:300, Abcam, ab150115) and goat anti‐rabbit IgG (1:200, Abcam, ab150077) secondary antibodies for 1 h. After DAPI staining for 10 min, cells were observed under the confocal laser.

### 2.10 Elisa

The levels of ROS (Solarbio, CA1410), SOD (Nanjing Jiancheng, A001‐3), and MDA (Nanjing Jiancheng, A003‐4‐1) in HCT116 cells were detected by commercial kits. To determine ROS levels, cells (1 × 10^6^ cells/mL) were incubated with 10 μM DCFH‐DA at 37°C for 20 min, and then washed three times with serum‐free medium. Fluorescence was recorded at 488/530 nm excitation/emission using a fluorescence microplate reader (MolecularDevices, SpectraMax M4). For SOD measurement, cells (1 × 10^6^) were homogenized in 0.3–0.5 mL of PBS. Subsequently, 20 μL of the sample was mixed with 20 μL of the enzyme working solution and 200 μL of the substrate solution, and then incubated at 37°C for 20 min. Absorbance at 450 nm was measured using a microplate reader. To assess MDA levels, cells were treated with 0.5 mL of the extraction solution for 2 min. Following this, 100 μL of the sample was mixed with 1 mL of working solution and incubated at 95°C for 40 min. After centrifugation, 0.25 mL of the reaction solution was added to the 96‐well plates, and absorbance at 530 nm was measured using a microplate reader.

### 2.11 Animals

Thirty BALB/c nude mice (6 weeks, 18–20 g) were purchased from Beijing Huafukang Biotechnology Co. Ltd. Mice were raised in a SPF‐grade room. To evaluate the tumor‐targeting ability of PPS‐ELNs, nude mice were randomly divided into the PBS and PPS‐ELNs groups, with 6 mice in each group. Mice were injected subcutaneously with 3 × 10^6^ HCT116 cells. When the tumor volume reached approximately 100 mm^3^, DiR‐labeled PPS‐ELNs (25 mg/kg) were injected into mice through the tail vein. Subsequently, the fluorescence distribution at 6, 12, and 24 h after injection was observed. CO_2_ euthanasia was conducted on animals, and heart, liver, spleen, lung, kidney, and tumor tissues were collected to assess the fluorescence distribution. To explore the anti‐CRC effects and mechanism of PPS‐ELNs, mice were divided into three groups (*n* = 6): control, PPS‐ELNs‐L (12.5 mg/kg), and PPS‐ELNs‐H (25 mg/kg). On day 7 after injection of HCT116 cells, PPS‐ELNs (12.5 or 25 mg/kg) were administered to mice via the tail vein. Drug treatment was given daily for 2 weeks, and the volume and weight of tumors were measured every 3 days. Tumor volume = (length × width^2^)/2. After 2 weeks of treatment, mice were euthanized and blood samples, major organs, and tumor tissues were collected. The animal procedures were performed in accordance with ARRIVE guidelines, and approved by the laboratory animal ethics committee of Yangzhou University (202509055).

### 2.12 TUNEL Staining

Tumor tissues were fixed with 4% paraformaldehyde for 48 h. Subsequently, the tissues were embedded in paraffin wax (Sinopharm, 69,019,061) and sectioned at a thickness of 4–7 μm. The apoptosis in tissue sections was detected using TUNEL apoptosis detection kit (Beyotime, C1086) according to the manufacturer's instructions. Sections were imaged under a fluorescence microscope (Olympus, CKX53).

### 2.13 Immunohistochemistry

Tumor tissue sections were placed in boiling citric acid repair solution (Beyotime, P0083) for high‐pressure repair and then treated with 3% H_2_O_2_ (Sinopharm, 10,011,208) for 20 min. Normal goat serum (50 μL, Solarbio, SL038) was added to sections for 10–15 min. Subsequently, sections were incubated with primary antibodies including Ki67 (1:300, Affinity, AF0198) and Gal3 (1:100, CST, 87985) at 4°C overnight. Next, sections were incubated with secondary antibody (1:2000, Abcam, ab205718) for 10–15 min. Following DAB (Beyotime, P0202) color development and hematoxylin (Solarbio, G1080) counterstaining, sections were observed under a microscope.

### 2.14 TEM

Tumor tissues and HCT116 cells were fixed in 2.5% glutaraldehyde (Solarbio, P1126) and then placed in 1% osmium tetroxide (TED PELLA INC, 18456) for 2 h. The tissues and cells were embedded in epoxy resin (TED PELLA INC, 18005) and cut into ultrathin sections of 70 nm using an ultramicrotome (Leica, EM UC7). After staining with uranyl acetate and lead citrate trihydrate (TED PELLA INC, 19312), sections were imaged by TEM.

### 2.15 Western Blot

Total protein was extracted from tumor tissues and cells using lysis buffer (Beyotime, P0013B), and protein concentration was measured by BCA method. Proteins were separated by SDS‐PAGE and transferred to PVDF membranes (Beyotime, FFP24). The blocked membranes were incubated with primary antibodies at 4°C overnight. Membranes were then incubated with secondary antibody (1:2000; Abcam, ab205718) for 1 h. Protein bands were developed by ECL (Applygen, P1000). The information of antibodies is as follows: Primary antibodies against Parkin (1:1000, AF0235), PINK1 (1:1000, DF7742), p62 (1:1000, AF5384), TOM20 (1:1000, AF5206), LC3 (1:1000, AF5402), GAPDH (1:3000, AF7021), Bax (1:1000, AF0120), and Bcl‐2 (1:1000, AF6139) were purchased from Affinity. Primary antibody against Gal3 (1:1000, 87,985) was purchased from CST.

### 2.16 HE Staining

Heart, liver, spleen, lung, and kidney tissues were fixed in 4% paraformaldehyde, embedded in paraffin, and sectioned at 4–7 μm thickness. Sections were stained with the HE staining kit (Beyotime, C0105S) and imaged under a microscope.

### 2.17 Biochemical Analysis

Serum alanine aminotransferase (ALT), aspartate aminotransferase (AST), creatinine, and urea levels were measured by a biochemical analyzer (Thermo Scientific, Indiko).

### 2.18 Statistics

Data are expressed as mean ± standard deviation. Unpaired *t*‐tests or one‐way analysis of variance followed by Tukey's post hoc test were employed to compare differences between groups (GraphPad Prism 7.0). *p* < 0.05 was considered statistically significant.

## 3 Results

### 3.1 PPS‐ELNs Inhibited Proliferation and Promoted Apoptosis in CRC Cells

**FIGURE 1:**
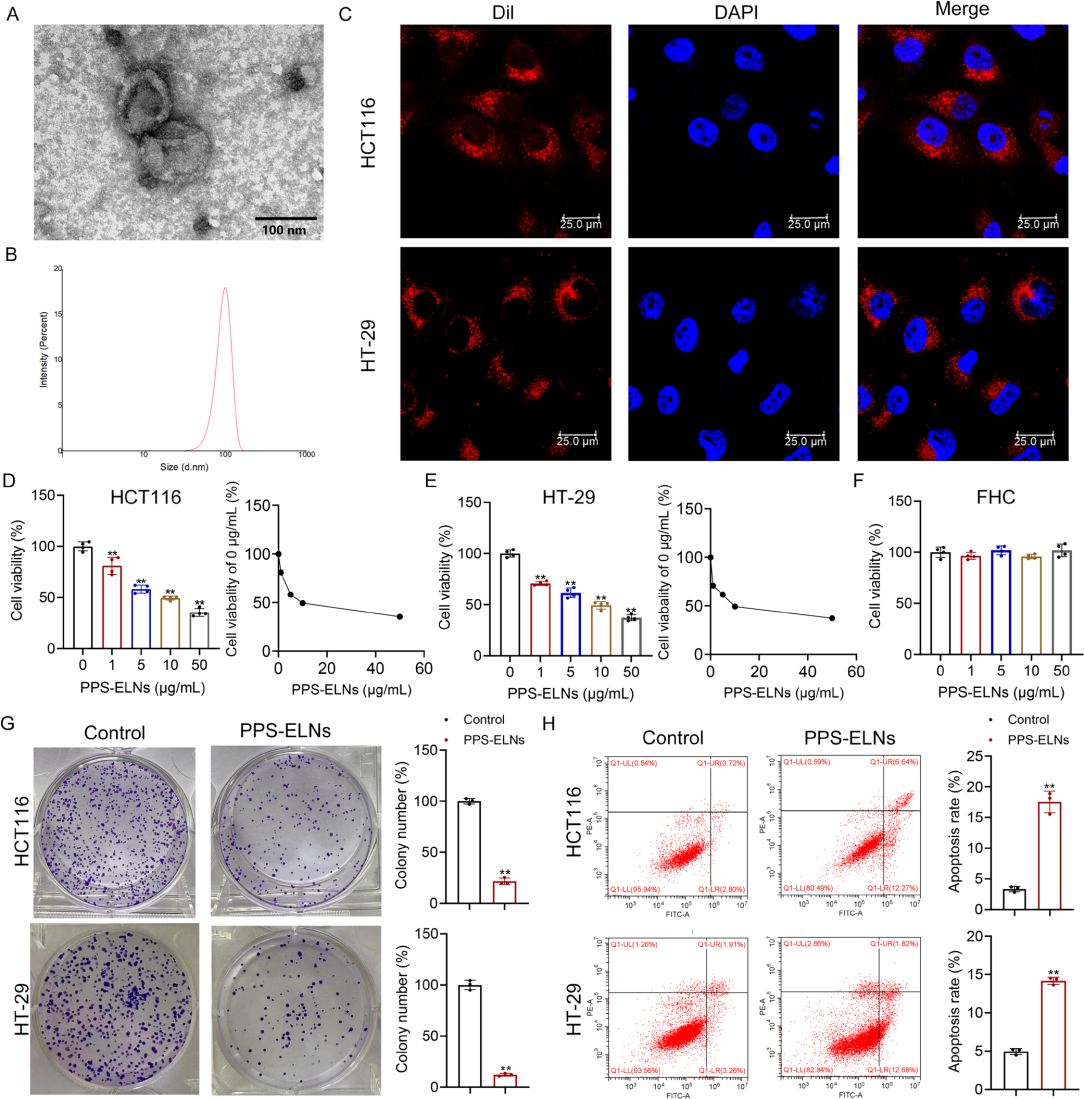
PPS‐ELNs inhibited proliferation and promoted apoptosis in CRC cells. (A) The morphology and structure of PPS‐ELNs were observed by TEM. Scale bar = 100 nm. (B) The particle size and zeta potential of PPS‐ELNs were analyzed using a Malvern Zetasizer. (C) Uptake of PPS‐ELNs by HCT116 and HT‐29 cells was assessed by laser confocal microscopy. Scale bar = 25 μm. The viability of HCT116 cells (D), HT‐29 cells (E), and FHC cells (F) was measured by CCK‐8 assay. (G) The proliferation of HCT116 and HT‐29 cells was assessed by colony formation assay. (H) Apoptosis rate in HCT116 and HT‐29 cells was detected by flow cytometry. **p < 0.01 vs. Control or 0 μg/mL group. PPS‐ELNs, *Pinellia pedatisecta* Schott‐derived exosome‐like nanovesicles; CRC, colorectal cancer; TEM, Transmission electron microscopy; CCK‐8, Cell counting kit‐8.

The prepared PPS‐ELNs had a spherical morphology and a double‐layer membrane structure (Figure 1A). Their particle size was 112.4 nm and zeta potential was −18.4 mV (Figure 1B). HCT116 and HT‐29 cells were co‐cultured with DiR‐labeled PPS‐ELNs to evaluate cellular uptake. Pronounced fluorescence signals were observed in both CRC cell lines (Figure 1C), suggesting efficient cellular uptake of PPS‐ELNs and supporting their potential as a delivery vector. Cell viability was measured using CCK‐8. Different doses of PPS‐ELNs notably decreased the viability of HCT116 and HT‐29 cells, with IC_50_ values of 11.77 and 12.18 μg/mL, respectively (Figure 1D,E). Therefore, 10 μg/mL PPS‐ELNs, a concentration close to the IC_50_ value, was applied for subsequent experiments to ensure sufficient efficacy while avoiding excessive nonspecific cytotoxicity. In addition, all tested doses of PPS‐ELNs did not affect the viability of FHC cells (Figure 1F), suggesting that PPS‐ELNs exhibit selective cytotoxicity for CRC cells over normal cells. Cell proliferation was assessed by colony formation assay. The results showed that the number of colonies in both HCT116 and HT‐29 cells in the PPS‐ELNs group was observably decreased compared to controls (Figure 1G). Moreover, the apoptosis rate of both cell types was markedly increased after PPS‐ELNs treatment (Figure 1H). These results indicate that PPS‐ELNs inhibit the proliferation of CRC cells and promote their apoptosis, demonstrating their potential anti‐cancer effects.

### 3.2 PPS‐ELNs Regulated Mitophagy Pathway in CRC Cells

**FIGURE 2:**
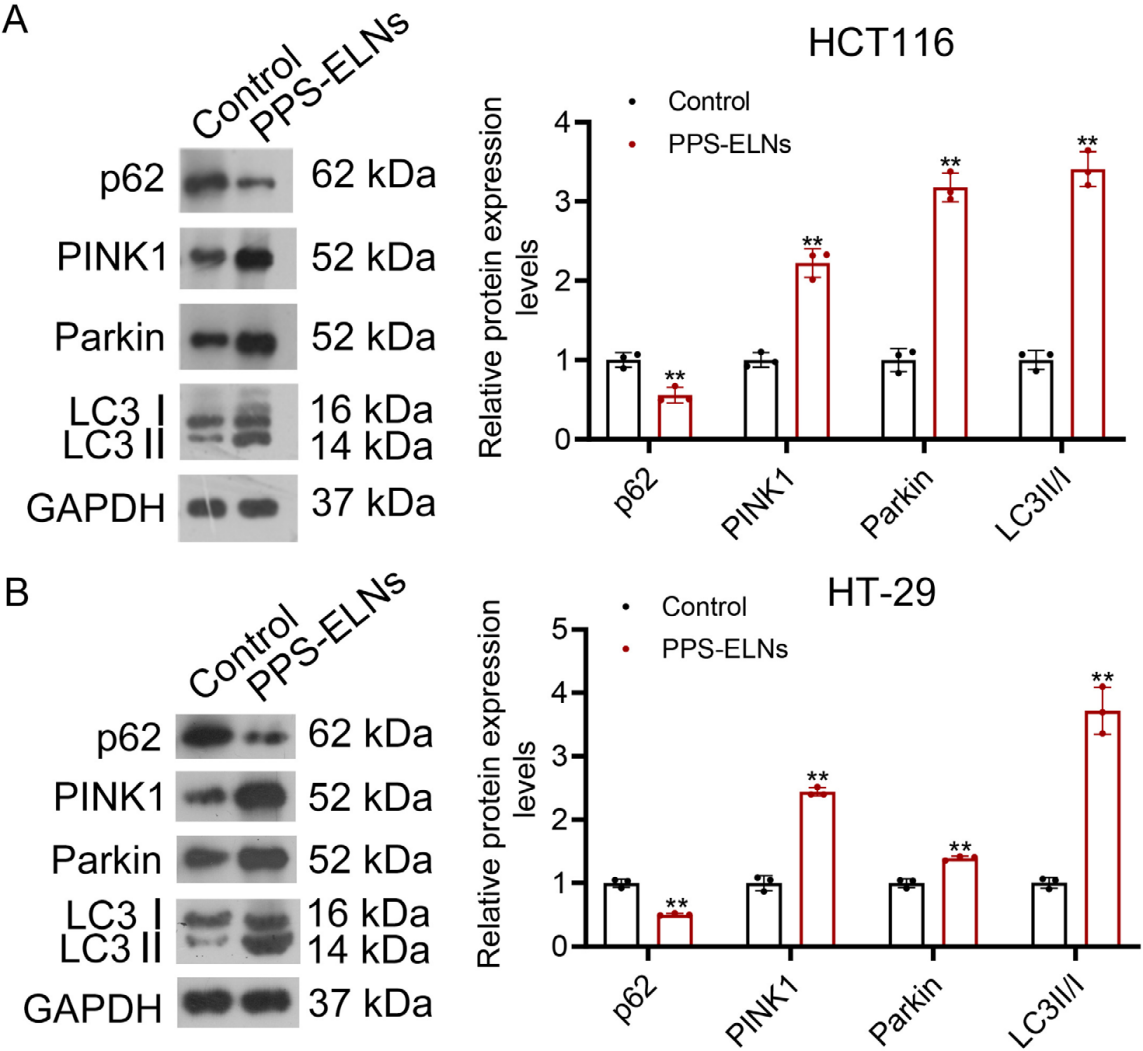
PPS‐ELNs regulated the mitophagy pathway in CRC cells. The expression of p62, PINK1, Parkin, and LC3‐II/I in HCT116 cells (A) and HT‐29 cells (B) was detected by Western blot. **p < 0.01 vs. Control group.

PINK1‐mediated mitophagy plays a tumor‐suppressing role in CRC (Arcos et al. 2025); therefore, we detected mitophagy pathway‐associated proteins by Western blot. Results showed that LC3II/I, PINK1, and Parkin levels were significantly increased, while p62 levels were decreased after PPS‐ELNs treatment in both HCT116 and HT‐29 cells (Figure 2A,B). Increased PINK1 and Parkin levels indicated activation of the mitophagy pathway, while elevated LC3II/I levels and decreased p62 levels reflected an enhanced autophagic flux. Altogether, the data demonstrate that PPS‐ELNs induce mitophagy in CRC cells, which may contribute to their anti‐CRC effects.

### 3.3 Lysosome Inhibitor Suppressed Mitophagy in PPS‐ELNs‐Treated HCT116 Cells

**FIGURE 3:**
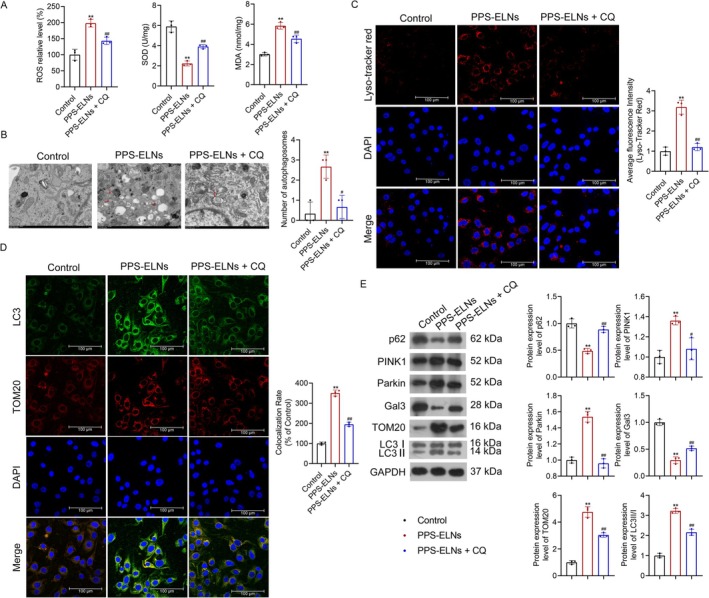
Lysosome inhibitor suppressed mitophagy in PPS‐ELNs‐treated HCT116 cells. (A) The levels of ROS, SOD, and MDA were detected by commercial kits. (B) Autophagosomes in HCT116 cells were observed by TEM. Scale bar = 1 μm. (C) Lysosomes in HCT116 cells were detected by LysoTracker Red staining. Scale bar = 100 μm. (D) The co‐localization of LC3 and mitochondrial protein TOM20 was detected by immunofluorescence. Scale bar = 100 μm. (E) The expression of p62, PINK1, Parkin, Gal3, TOM20, and LC3‐II/I was detected by Western blot. **p < 0.01 vs. Control group. ^#^ *p* < 0.05, ^##^ *p* < 0.01 vs. PPS‐ELNs group. CQ, chloroquine.

To determine the involvement of lysosome‐mediated mitophagy in PPS‐ELNs treatment of CRC, HCT116 cells were treated with CQ, a lysosome inhibitor. Given that oxidative stress‐induced mitochondrial damage is the primary trigger for mitophagy, oxidative stress markers were detected by commercial kits. ROS and MDA levels were observably elevated after PPS‐ELNs treatment, but SOD showed the opposite trend, and CQ reversed these changes (Figure 3A). This result suggests that PPS‐ELNs induce oxidative stress, which may be involved in lysosome‐mediated mitophagy. Accordingly, further experiments were conducted to assess changes in lysosome‐mediated mitophagy and its modulation by CQ. TEM results revealed that PPS‐ELNs notably increased the number of autophagosomes in HCT116 cells, but this number decreased after CQ treatment (Figure 3B). Lysosomes were detected by LysoTracker Red staining. Results demonstrated that the number of lysosomes in the PPS‐ELNs group was significantly increased compared to controls, while CQ treatment decreased their number (Figure 3C). Additionally, immunofluorescence results showed that LC3 and mitochondrial protein TOM20 were co‐localized, and their levels in HCT116 cells were observably increased after PPS‐ELNs treatment, which was inhibited by CQ (Figure 3D). PINK1, Parkin, TOM20, and LC3II/I levels were significantly increased, while p62 and Gal3 levels were decreased after PPS‐ELNs treatment, and these effects were reversed by CQ (Figure 3E). Taken together, the above data indicate that lysosome‐mediated mitophagy is activated following PPS‐ELNs treatment but is inhibited by CQ. This suggests that lysosome‐mediated mitophagy may be involved in the anti‐CRC mechanism of PPS‐ELNs.

### 3.4 PPS‐ELNs Inhibited CRC Progression by Upregulating the Lysosome‐Mediated Mitophagy Pathway

**FIGURE 4:**
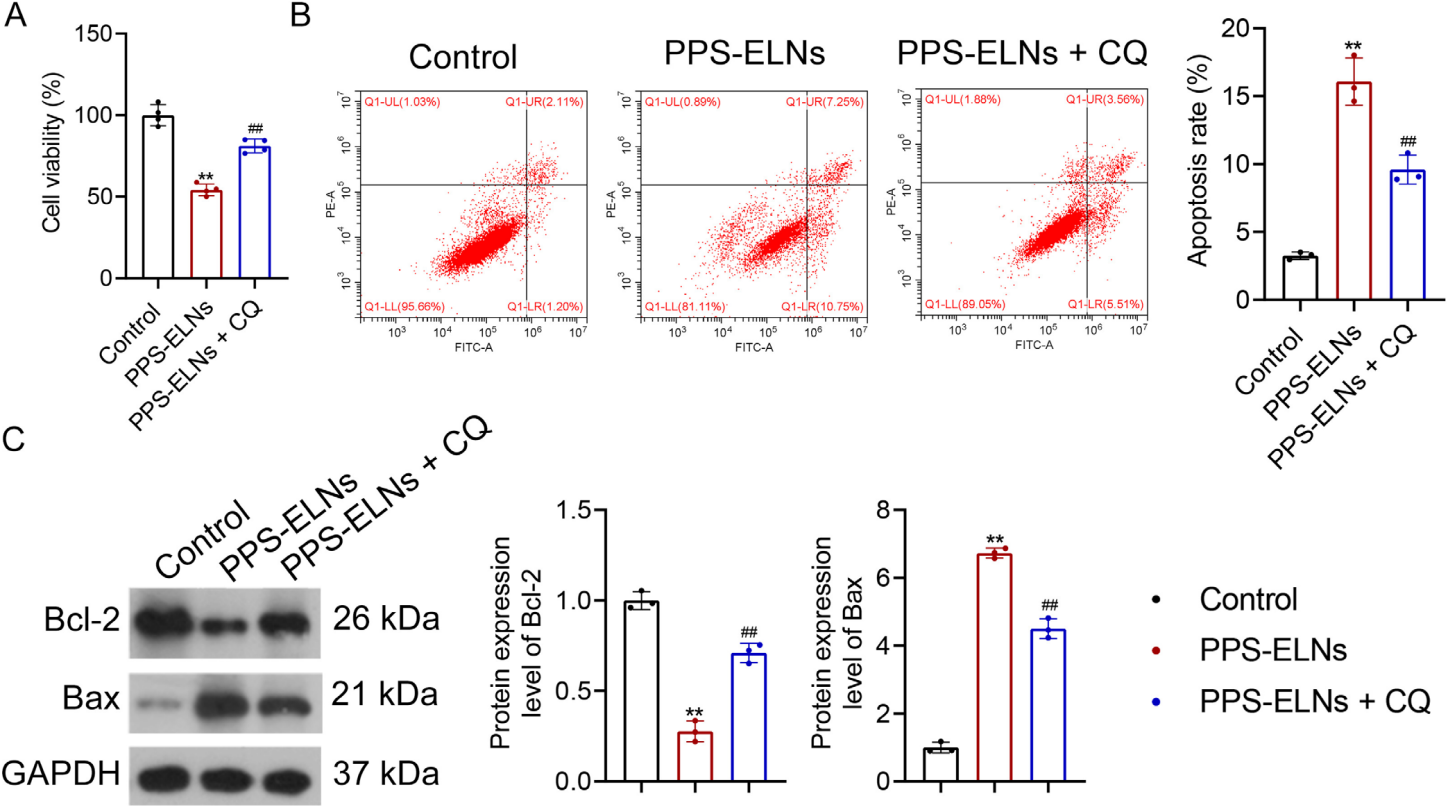
PPS‐ELNs inhibited CRC progression by upregulating the lysosome‐mediated mitophagy pathway. (A) The viability of HCT116 cells was measured by CCK‐8 assay. (B) The apoptosis rate in HCT116 cells was detected by flow cytometry. (C) The expression of Bcl‐2 and Bax was detected by Western blot. **p < 0.01 vs. Control group. ^##^ *p* < 0.01 vs. PPS‐ELNs group.

Based on the above findings, the effects of PPS‐ELNs on the proliferation and apoptosis of CRC cells were further investigated in the presence of CQ. PPS‐ELNs markedly reduced the viability of HCT116 cells, but CQ treatment partially restored it (Figure 4A). Moreover, apoptosis in the PPS‐ELNs group was notably increased compared to controls, which was reversed by CQ treatment (Figure 4B). A significant decrease in anti‐apoptotic protein Bcl‐2 expression and a notable increase in pro‐apoptotic protein Bax expression were observed after PPS‐ELNs treatment, but CQ treatment altered these protein levels (Figure 4C). These findings suggest that PPS‐ELNs exert anti‐CRC effects, at least in part, by regulating lysosome‐mediated mitophagy.

### 3.5 PPS‐ELNs Inhibited CRC Progression In Vivo

**FIGURE 5:**
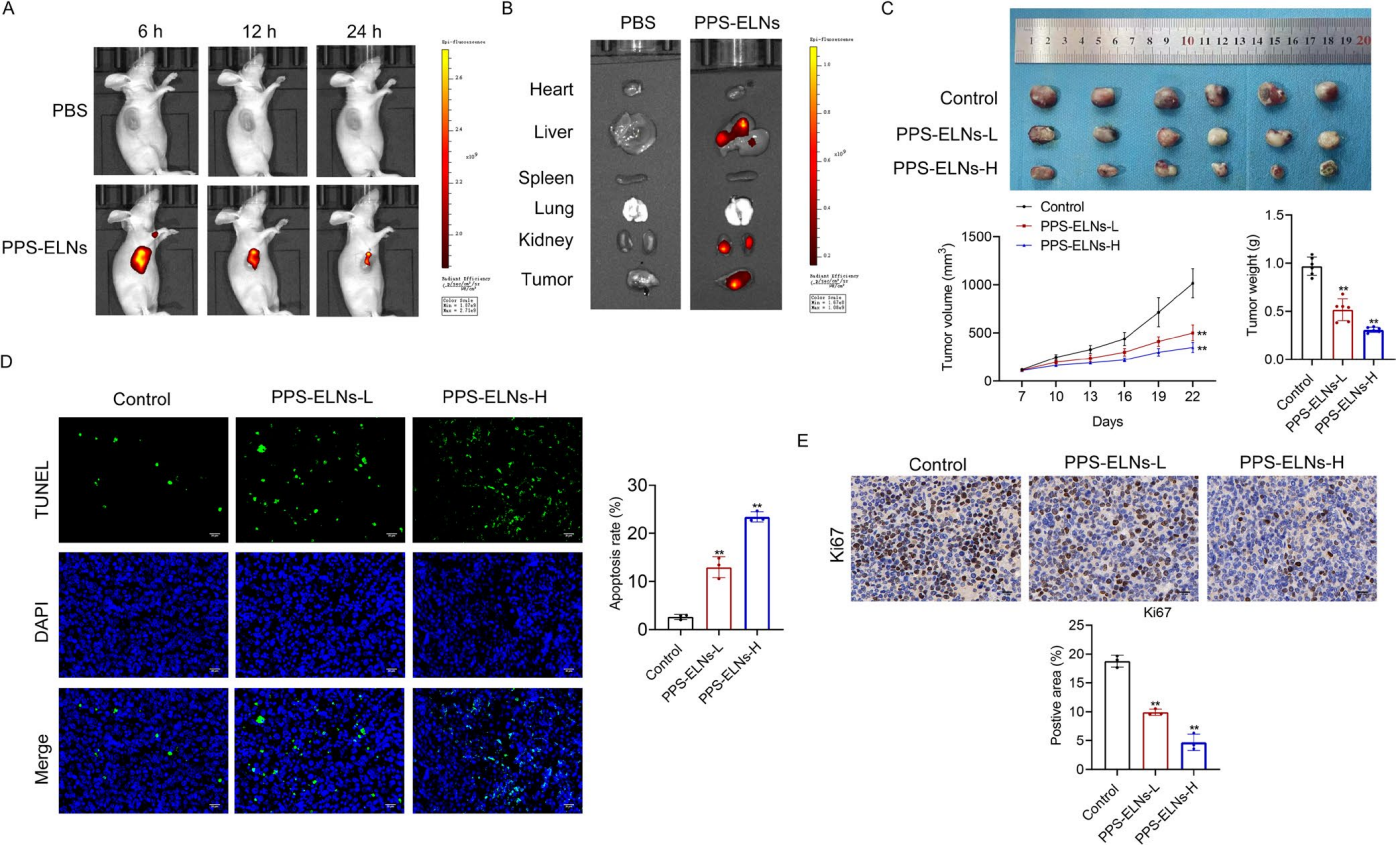
PPS‐ELNs inhibited tumor progression in CRC mice. (A) Representative images of the fluorescence distribution in nude mice at 6, 12, and 24 h after injection of DiR‐labeled PPS‐ELNs. (B) Representative images of in vitro fluorescence signals in major organs (heart, liver, spleen, lung, and kidney) and tumor tissues at 24 h after injection of DiR‐labeled PPS‐ELNs. (C) Tumor volume and weight of mice. (D) Apoptosis rate in tumor tissues was detected by TUNEL staining. Scale bar = 20 μm. (E) The expression of Ki67 in tumors was detected by immunohistochemistry. Scale bar = 20 μm. **p < 0.01 vs. Control group.

To evaluate the biodistribution of PPS‐ELNs in vivo, DiR‐labeled PPS‐ELNs were injected into CRC nude mice. In vivo imaging demonstrated that no fluorescence was observed in the PBS group, while in the PPS‐ELNs group, the fluorescence gradually increased over time and reached its maximum intensity at 6 h (Figure 5A). The fluorescence eventually concentrated in mouse tumors, which indicated the tumor‐targeting potential of PPS‐ELNs. After 24 h of PPS‐ELNs injection, the fluorescence in the isolated organs was concentrated in the liver, kidney, and tumor (Figure 5B), suggesting that the liver and kidneys might be the primary organs for the distribution and clearance of PPS‐ELNs. The above observations collectively support the potential of PPS‐ELNs as a delivery vector for targeted CRC therapy in vivo.

Subsequently, we further evaluated the therapeutic effect of PPS‐ELNs on CRC mice. Tumor volume and weight of control mice were markedly reduced following PPS‐ELNs treatment (Figure 5C). Apoptosis in tumors was assessed using TUNEL staining. Results demonstrated that apoptosis of control mice was notably increased after treatment with PPS‐ELNs (Figure 5D). Ki67 expression in tumor tissues was detected by immunohistochemistry. The results showed that PPS‐ELNs significantly decreased Ki67 expression in control mice (Figure 5E), indicating inhibition of cell proliferation. These findings demonstrate that PPS‐ELNs can effectively inhibit tumor progression in CRC mice by promoting apoptosis and suppressing proliferation.

### 3.6 PPS‐ELNs Regulated Lysosome‐Mediated Mitophagy Pathway in CRC Mice

**FIGURE 6:**
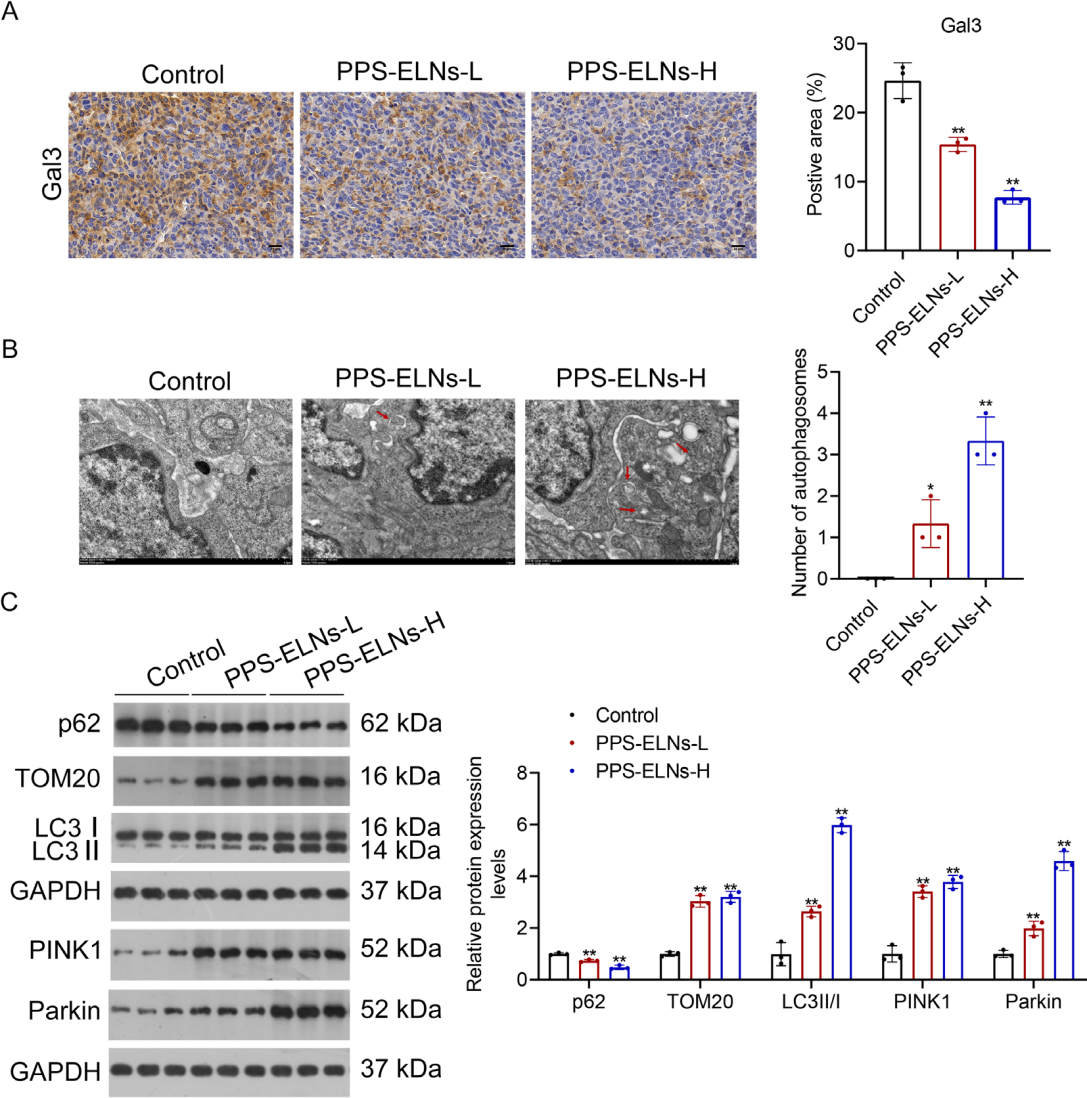
PPS‐ELNs regulated lysosome‐mediated mitophagy pathway in CRC mice. (A) The expression of Gal3 in tumors was detected by immunohistochemistry. Scale bar = 20 μm. (B) Autophagosomes in tumor tissues were observed by TEM. Scale bar = 1 μm. (C) The expression of p62, TOM20, LC3‐II/I, PINK1, and Parkin was detected by Western blot. *p < 0.05, **p < 0.01 vs. Control group.

The role of the lysosome‐mediated mitophagy pathway in PPS‐ELNs‐treated CRC mice was assessed. Consistent with the observations in vitro, PPS‐ELNs markedly downregulated Gal3 expression in control mice (Figure 6A). Autophagosomes in tumor tissues were detected by TEM. Results demonstrated an increase in autophagosome numbers in control mice after PPS‐ELNs treatment (Figure 6B). Mitophagy pathway‐related proteins were analyzed by Western blot. The results displayed that PPS‐ELNs treatment significantly increased LC3II/I, TOM20, PINK1, and Parkin levels in CRC mice, while p62 showed an opposite trend (Figure 6C). These results indicate that PPS‐ELNs induce lysosome‐mediated mitophagy in CRC mice, which may be the key mechanism by which they exert their anti‐cancer effects.

### 3.7 PPS‐ELNs Demonstrated Favorable Biocompatibility In Vivo

**FIGURE 7:**
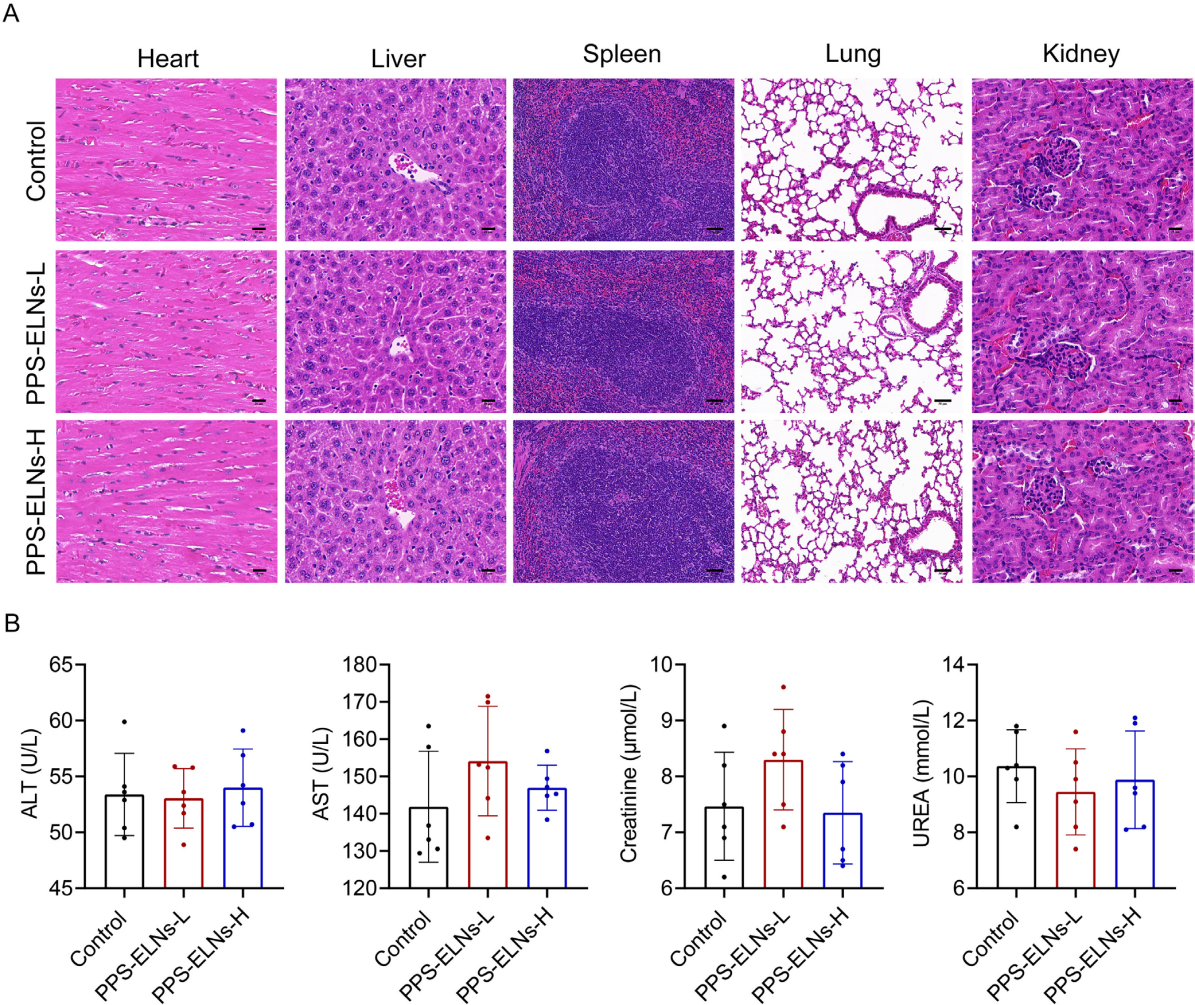
PPS‐ELNs demonstrated favorable biocompatibility in vivo. (A) Representative images of HE staining of heart, liver, spleen, lung, and kidney tissues to assess biocompatibility. (B) Serum ALT, AST, creatinine, and UREA levels were detected by a biochemical analyzer. ALT, alanine aminotransferase; AST, aspartate aminotransferase.

Organ tissues were examined by HE staining to evaluate biocompatibility. The results showed no significant damage to the heart, liver, spleen, lung, or kidney in mice treated with PPS‐ELNs (Figure 7A), which suggests the low toxicity of PPS‐ELNs. Moreover, PPS‐ELNs treatment had no notable effects on the levels of ALT, AST, creatinine, and UREA in the control mice (Figure 7B), demonstrating that PPS‐ELNs do not impair liver or kidney function. These findings indicate the favorable safety profile of PPS‐ELNs.

## 4 Discussion

CRC is a common malignant tumor with high morbidity and mortality (Bray et al. 2024). Our study revealed that PPS‐ELNs suppressed tumor growth in CRC mice. Additionally, PPS‐ELNs reduced tumor cell proliferation and promoted apoptosis. Furthermore, PPS‐ELNs reduced oxidative stress, alleviated lysosomal damage, and induced mitophagy. Treatment with lysosome inhibitor CQ reversed the effect of PPS‐ELNs. Therefore, this study suggested that PPS‐ELNs exerted an anti‐CRC effect through the lysosome‐mediated mitophagy pathway. Notably, PPS‐ELNs demonstrated excellent biocompatibility, highlighting their potential for clinical translation.

During the development of CRC, cancer cells gradually erode the intestinal wall and replace healthy intestinal tissue, and the tumor microenvironment (TME) further promotes the proliferation and survival of CRC cells (Urbaniec‐Stompór et al. 2024). Studies have shown that medicinal plant‐derived ELNs can regulate the TME through various drug delivery routes and exert anti‐tumor effects (Feng, Huang, et al. 2025b). *Citrus limon* ‐derived ELNs inhibit the growth of CRC, partially through the regulation of lipid metabolism (Raimondo et al. 2018). The rhizome of PPS is a well‐known TCM with various biological activities, including antiemetic, anti‐inflammatory, and antiviral properties (Peng et al. 2022). Previous studies have shown that PPS extract can affect the immune microenvironment of HPVTC‐1 tumors, directly inhibiting tumor growth (Huang et al. 2018). We prepared PPS‐ELNs and revealed that they significantly inhibited tumor growth in CRC mice and reduced the viability of CRC cell lines. Ki67 is a cell proliferation marker, and its positive staining in tumor tissues is employed to grade primary tumors (Remnant et al. 2021). Ki67 expression was increased in CRC mice, but hirsutine treatment decreased its expression, thereby alleviating CRC (Ren et al. 2024). Similarly, our results demonstrated that Ki67 expression was notably reduced after PPS‐ELNs treatment. Moreover, cell experiments revealed that PPS‐ELNs effectively inhibited cell proliferation.

Programmed cell death, such as apoptosis, autophagy, and pyroptosis, plays an important role in maintaining homeostasis, which can clear damaged cells at risk of tumorigenesis (Taha et al. 2025). Research has shown that overexpression of RNF112 induces apoptosis and inhibits CRC development (Li et al. 2025). Illudin S treatment induces apoptosis in CRC cells, prolongs the survival of CRC mice, and prevents tumor progression (Lee et al. 2025). Another study demonstrated that PPS extract promotes the apoptosis of leukemia cells in mice (Li et al. 2023). Similarly, we found that PPS‐ELNs promoted apoptosis in CRC tissue and cells. Mitochondria are key organelles of eukaryotic cells, involved in cellular metabolism, programmed cell death, and TME. Mitochondrial dysfunction of tumor and immune cells in the TME is closely associated with cancer progression (Wang et al. 2024). Mitophagy degrades damaged mitochondria through autophagy mechanisms to ensure cell homeostasis (Zhu et al. 2024). Wu et al. (2024) revealed that USP26 induces CRC by downregulating PRKN‐mediated mitophagy. Research has shown that Tong‐Xie‐Yao‐FANG promotes mitophagy in colon epithelial cells to improve CRC via PINK1/Parkin pathway (Xu et al. 2024). Similarly, we found that PPS‐ELNs increased the number of autophagosomes and altered mitophagy‐related protein levels in CRC models. Mitochondria and lysosomal compartments are closely interrelated, and the connections between them involve mitophagy, organelle dynamics, and various signaling cascades (Lang et al. 2022). Activation of lysosomal ROS‐sensitive Ca^2+^ channels reduces mitochondrial damage and ROS levels, promoting lysosomal biogenesis and mitophagy (Feng, Cai, et al. 2025a). We found that PPS‐ELNs induced oxidative stress, decreased the expression of Gal3 (a marker of lysosome damage), and increased lysosome stability. PPS‐ELNs' therapeutic effect on CRC may involve lysosome‐mediated mitophagy pathway. However, treatment with the lysosome inhibitor CQ inhibited the lysosome‐mediated mitophagy pathway and altered the therapeutic effect of PPS‐ELNs on CRC.

However, this study still has some limitations. Firstly, lysosome inhibitors have not been applied in CRC mice, and more experiments are needed to clarify the in vivo mechanism. Additionally, PPS‐ELNs contain various active ingredients, which may contribute to multiple mechanisms. Therefore, further investigation is needed to fully elucidate the role of PPS‐ELNs in CRC.

## 5 Conclusion

This study demonstrates that PPS‐ELNs are efficiently taken up by CRC cells and exhibit tumor‐targeting properties in vivo. They inhibit CRC cell proliferation and promote apoptosis, thereby suppressing tumor progression. Mechanistically, PPS‐ELNs exert their anti‐cancer effects by regulating the lysosome‐mediated mitophagy pathway. Importantly, PPS‐ELNs display minimal cytotoxicity in normal cells and have a favorable safety profile in vivo. These findings offer novel perspectives for CRC treatment.

## Ethics Statement

All experiments adhered to the guidelines approved by the Ethical Review Committee for Animal Experiments of Yangzhou University (202509055). In addition, all methods were carried out in accordance with the relevant guidelines and regulations.

## Conflicts of Interest

The authors declare no conflicts of interest.

## Data Availability

The presented data is available upon request from the corresponding authors.
